# Antibody-based PET of uPA/uPAR signaling with broad applicability for cancer imaging

**DOI:** 10.18632/oncotarget.12528

**Published:** 2016-10-08

**Authors:** Dongzhi Yang, Gregory W. Severin, Casey A. Dougherty, Rachel Lombardi, Daiqin Chen, Marcian E. Van Dort, Todd E. Barnhart, Brian D. Ross, Andrew P. Mazar, Hao Hong

**Affiliations:** ^1^ Center for Molecular Imaging, Department of Radiology, University of Michigan, Ann Arbor, Michigan 48109, United States; ^2^ Jiangsu Key Laboratory of New Drug Research and Clinical Pharmacy, Xuzhou Medical University, Xuzhou, Jiangsu 221004, China; ^3^ Center for Nuclear Technologies, Technical University of Denmark, 4000 Roskilde, Denmark; ^4^ Department of Chemistry, Facility for Rare Isotope Beams, Michigan State University, East Lansing, Michigan 48824, United States; ^5^ Department of Chemistry, Iona College, New Rochelle, New York 10801, United States; ^6^ Department of Medical Physics, University of Wisconsin-Madison, Madison, Wisconsin 53705, United States; ^7^ Department of Pharmacology, Feinberg School of Medicine, Northwestern University, Chicago, Illinois 60611, United States

**Keywords:** urokinase plasminogen activator (uPA), uPAR, immunoPET, quantitative analysis, cancer biomarkers

## Abstract

Mounting evidence suggests that the urokinase plasminogen activator (uPA) and its receptor (uPAR) play a central role in tumor progression. The goal of this study was to develop an ^89^Zr-labeled, antibody-based positron emission tomography (PET) tracer for quantitative imaging of the uPA/uPAR system. An anti-uPA monoclonal antibody (ATN-291) was conjugated with a deferoxamine (Df) derivative and subsequently labeled with ^89^Zr. Flow cytometry, microscopy studies, and competitive binding assays were conducted to validate the binding specificity of Df-ATN-291 against uPA. PET imaging with ^89^Zr-Df-ATN-291 was carried out in different tumors with distinct expression levels of uPA. Biodistribution, histology examination, and Western blotting were performed to correlate tumor uptake with uPA or uPAR expression. ATN-291 retained uPA binding affinity and specificity after Df conjugation. ^89^Zr-labeling of ATN-291 was achieved in good radiochemical yield and high specific activity. Serial PET imaging demonstrated that, in most tumors studied (except uPA- LNCaP), the uptake of ^89^Zr-Df-ATN-291 was higher compared to major organs at 120 h post-injection, providing excellent tumor contrast. The tumor-to-muscle ratio of ^89^Zr-Df-ATN-291 in U87MG was as high as 45.2 ± 9.0 at 120 h p.i. In vivo uPA specificity of ^89^Zr-Df-ATN-291 was confirmed by successful pharmacological blocking of tumor uptake with ATN-291 in U87MG tumors. Although the detailed mechanisms behind *in vivo* ^89^Zr-Df-ATN-291 tumor uptake remained to be further elucidated, quantitative PET imaging with ^89^Zr-Df-ATN-291 in tumors can facilitate oncologists to adopt more relevant cancer treatment planning.

## INTRODUCTION

Numerous cellular events (e.g. cell migration, invasion, angiogenesis etc.) orchestrate the initiation, progression, and metastasis of cancer [1]. Over the past several decades, a multitude of biomarkers have been identified with the goal of elucidating these events for use as diagnostic/therapeutic targets. Among these markers, the urokinase plasminogen activator (uPA) system, which is primarily composed of uPA (a serine protease), uPA receptors (uPAR), and several inhibitors/modulators of uPA (especially plasminogen activator inhibitor 1 [PAI-1]), have attracted special attention as they serve as fundamental mediators of signaling pathways contributing to tumor microenvironment regulation [2]. Most important of all, accumulating evidence suggests a central role of uPA/uPAR in cancer progression especially in cancer metastasis [3]. uPA along with its “partners” (e.g. PAI-1) can serve as useful biomarkers to forecast disease progression as their expression levels in tumor tissues correlates with prognosis in different types of cancer [4]. Expression of uPA and uPAR has been confirmed in almost every solid tumor type examined, as well as in certain hematologic malignancies [5].

As a serine protease, the structure of uPA is formed by three distinct regions: a growth factor-like domain (GFD), a kringle domain, and a serine protease domain [6]. uPA possesses very specific proteolytic activity against its preferred substrate, plasminogen, and catalyzes its activation to plasmin. This transformation triggers the activation of various proteases and causes the degradation of the extracellular matrix (ECM) [7], eventually increasing cell motility, invasion, and survival [8]. The interaction between uPA and uPAR recruits uPA to the cell surface and is known to enhance its catalytic efficiency for plasminogen activation by up to two orders of magnitude [9].

ATN-291 is an IgG1 monoclonal antibody which binds specifically to the kringle domain of human uPA with a dissociation constant (K_d_) of ~0.5 nM, and it does not cross-react with murine uPA. After interaction with uPA, ATN-291 is internalized into cells via a uPA/uPAR specific manner and the status of uPA (activated or complexed with PAI-1) does not interfere with its binding to ATN-291 [10, 11]. Due to its high selectivity against uPA, ATN-291 was conjugated onto stealth liposomes (named nanobins) and demonstrated enhanced cargo delivery and therapeutic efficacy in ovarian cancer murine xenografts [12]. We primarily selected ATN-291 as a candidate for PET imaging development as it could provide real-time global monitoring of uPA/uPAR expression as a whole (instead of specifically targeting only uPA), which could be more relevant for cancer diagnosis and therapeutic response evaluation.

Due to the aforementioned important role of uPA/uPAR, much research attention has been devoted to the development of different relevant imaging/diagnostic agents, particularly targeting uPAR [13]. Among these, a well-known example is AE105 (screened from phage display library, composed of 9 amino acids in core structure), a peptide antagonist for uPA/uPAR interaction [13]. AE105 derivatives are applicable for intra-operative guidance during cancer surgery [14] and also for evaluation of cancer aggressiveness in human patients [15, 16]. Antibodies represent another attractive category of uPAR-targeted agents. With more potent, selective and sustained uptake in tumors, these antibody derivatives against uPAR could be used for tumor detection, surgery guidance, and drug resistance screening [17, 18]. Compared with uPAR-targeted agents, studies on uPA-selective agents are relatively limited. Representative examples include an ^18^F-labeled small molecule inhibitor used for targeting of breast cancer *in vivo* [19], and more recently, an ^111^In-labeled antibody adopted for prostate cancer imaging with remarkable tumor accumulation [20].

Our goal was to investigate a novel probe for effective targeting and imaging of the uPA/uPAR system in cancer with excellent targeting specificity and image contrast. To achieve this goal, ^89^Zr-labeled ATN-291 (i.e., ^89^Zr-Df-ATN-291; Df is abbreviated for deferoxamine) was used as an immunoPET probe. ^89^Zr (t_1/2_ = 78.4 h) was selected as the radiolabel in this study to provide a longitudinal evaluation on the interaction between ATN-291 and different tumor types [21]. To accomplish this goal, various *in vitro*, *in vivo*, and *ex vivo* studies were carried out to assess the binding of ^89^Zr-Df-ATN-291 to uPA in five tumor types (breast, prostate, ovarian, pancreatic, and glioblastoma). To the best of our knowledge, this is the first report on immuoPET imaging of uPA in cancer.

## RESULTS

### *In vitro* investigation of Df-ATN-291

**Figure 1:**
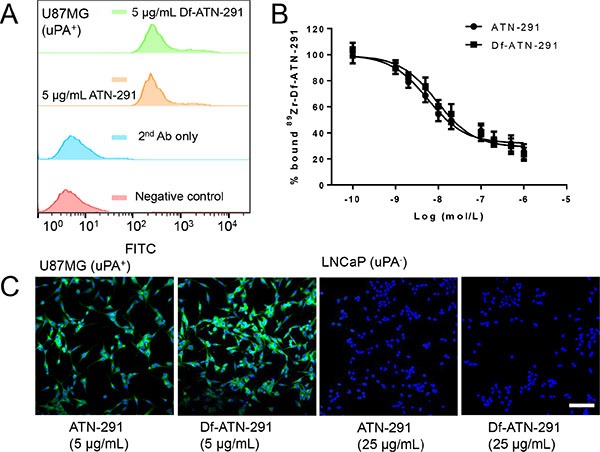
*In vitro* investigation of Df-ATN-291 (**A**) Flow cytometry analysis of ATN-291 and Df-ATN-291 in U87MG (high uPA) cells at the concentration of 5 μg/mL. (**B**) Competitive cell binding assay in U87MG demonstrated that Df-ATN-291 binds to cellular uPA with similar affinity to ATN-291. (**C**) Confocal fluorescence microscopy images of U87MG and LNCaP cells (low uPA) stained by ATN-291 or Df-ATN-291 (5 μg/mL in U87MG, and 25 μg/mL in LNCaP) followed by FITC-labeled secondary antibody. Scale bar: 50 μm.

Before initiating *in vivo* studies, we confirmed that the uPA binding activity and specificity of ATN-291 is not compromised after conjugation of Df. Data from FACS analysis of U87MG, which expresses high levels of uPA, suggests no observable differences in binding activity for cellular uPA between ATN-291 and Df-ATN-291 at the concentration of 5 μg/mL (Figure 1A). The competitive binding assay carried out in U87MG cells further confirmed similar uPA (IC_50_: 5.7 nM for ATN-291, 9.2 nM for Df-ATN-291) affinity between ATN-291 and Df-ATN-291 (Figure 1B). Fluorescence microscopy examination was performed in U87MG (uPA^+^) and LNCaP (uPA^−^) cells. In comparison with the potent accumulation in U87MG, both ATN-291 and Df-ATN-291 demonstrated minimal interaction with LNCaP cells (low uPA expression) even at the much higher concentration of 25 μg/mL (Figure 1C), which confirmed its antigen specificity. Taken together, these *in vitro* studies confirms that Df conjugation did not cause a significant alteration on the antigen-binding capacity or specificity of ATN-291.

### Radiochemistry

The ^89^Zr-labeling procedure, including final purification using PD-10 columns, was completed in 120 ± 15 min (*n* = 7). The decay-corrected radiochemical yield was 51 ± 11%, a result based on the calculation of 30 μg of Df-ATN-291 per 37 MBq of ^89^Zr and an estimated ^89^Zr-Df-ATN-291 specific activity of 0.63 GBq/mg antibody (assuming full protein recovery after PD-10). The radiochemical purity of ^89^Zr-Df-ATN-291 was > 98% based on decay-corrected ITLC readings.

### PET imaging in U87MG tumor-bearing mice

**Figure 2:**
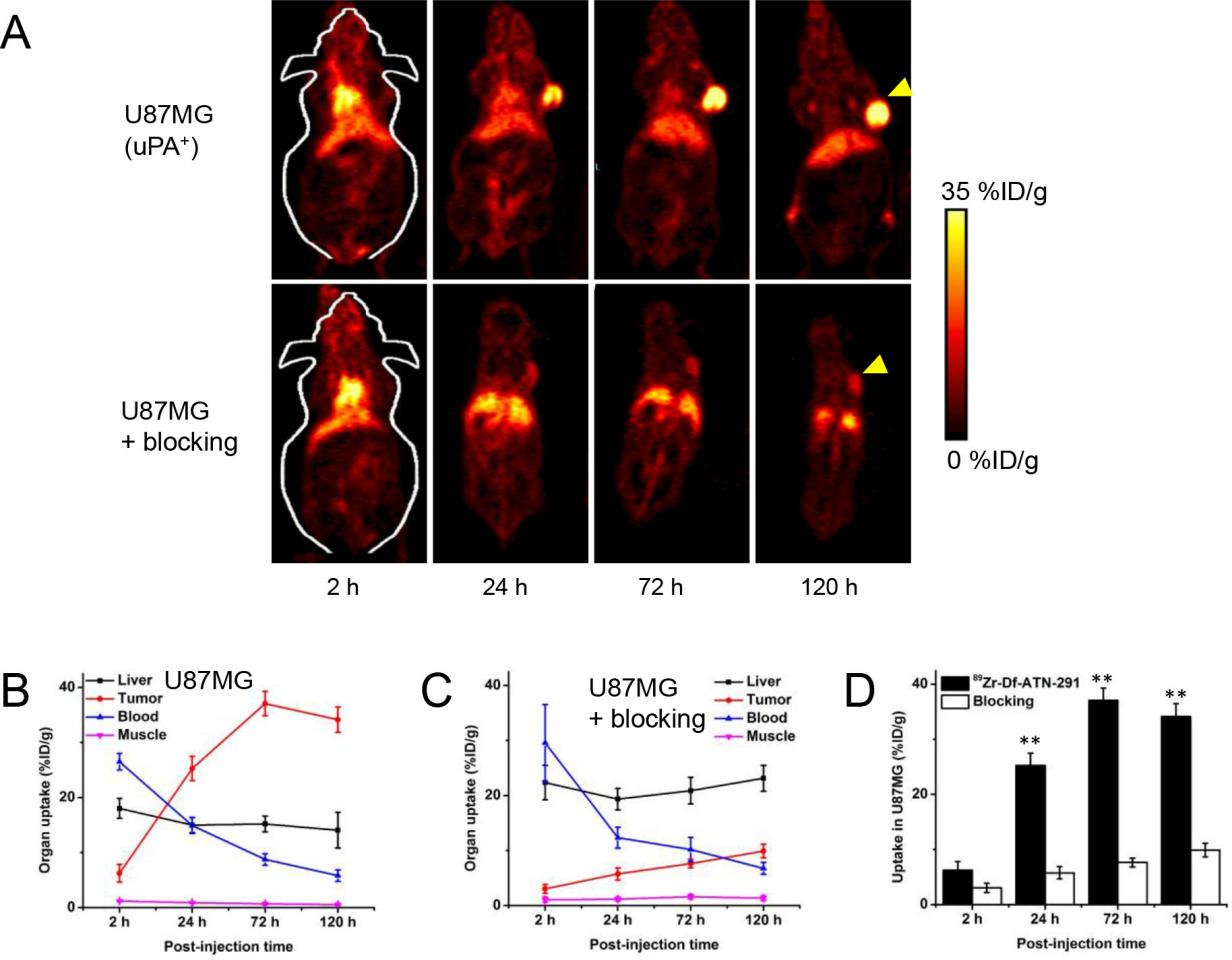
*In vivo* PET studies in U87MG tumor bearing mice (**A**) Representative PET images (coronal slice containing the center of the tumor) of U87MG tumor-bearing mice at 2, 24, 72, and 120 h post-injection of ^89^Zr-Df-ATN-291 and ATN-291 (~40 mg/kg) before ^89^Zr-Df-ATN-291 (i.e. blocking). Yellow arrowheads indicate the location of tumors. Time-activity curves of U87MG tumor, liver, blood, and muscle are also shown upon injection of (**B**) ^89^Zr-Df-ATN-291 (*n* = 4) and (**C**) ^89^Zr-Df-ATN-291 with blocking (*n* = 3). (**D**) Comparison of U78MG tumor uptake in ^89^Zr-Df-ATN-291 and blocking group. **P < 0.01.

Due to the extended *in vivo* circulation time of antibodies based on our previous experience [22–24], all PET imaging was conducted at 2, 24, 72, and 120 h after intravenous injection of the tracer. The circulation half-life of ^89^Zr-Df-ATN-291 was determined to be 11.9 ± 3.5 h based on sequential blood sampling from tail vein (Supplementary Figure S1). To initially determine the pharmacokinetics of ^89^Zr-Df-ATN-291, serial PET scans in U87MG (with known overexpression of uPA [25]) tumor-bearing mice (*n* = 4) were carried out. Reconstructed coronal slices that contained the U87MG tumors are shown in Figure 2A. From the Figure, we can observe that ^89^Zr-Df-ATN-291 was primarily retained in the blood pool at an early time point (2 h p.i.) and gradually cleared thereafter (Figure 2B), while its uptake in liver displayed the same trend (Figure 2B and Table 1). The accumulation of ^89^Zr-Df-ATN-291 in U87MG tumor increased over time (clearly visible at 24 h p.i.) and plateaued at around 72 h p.i., with the uptake being 6.2 ± 1.6, 25.3 ± 2.2, 37.1 ± 2.2, and 34.1 ± 2.3%ID/g at 2, 24, 72, and 120 h p.i., respectively (Figure 2A and 2B, Table 1). No significant renal clearance and bone accumulation was observed for ^89^Zr-Df-ATN-291, which further demonstrated the integrity of ^89^Zr-Df-ATN-291 *in vivo* within the time frame of PET imaging [26].

**Table 1:** Tissue uptakes of ^89^Zr-Df-ATN-291 in U87MG tumor-bearing mice and blocking group (n = 4 for U87MG group, and n = 3 for blocking group)

	Tumor (%ID/g)	Liver (%ID/g)	Blood (%ID/g)	Muscle (%ID/g)	Tumor-to-muscle
**2 h p.i.**					
**U87MG**	6.2 ± 1.6	18.0 ± 1.8	26.5 ± 1.5	1.1 ± 0.2	4.2 ± 1.2
**Blocking**	3.0 ± 0.8	22.3 ± 3.1	29.6 ± 7.0	1.1 ± 0.5	3.1 ± 0.8
**24 h p.i.**					
**U87MG**	25.3 ± 2.2	15.0 ± 1.4	15.0 ± 1.4	0.9 ± 0.2	22.6 ± 4.4
**Blocking**	5.8 ± 1.1	19.3 ± 2.0	12.3 ± 1.9	1.2 ± 0.4	5.1 ± 1.4
**72 h p.i.**					
**U87MG**	37.1 ± 2.2	15.2 ± 1.4	8.8 ± 1.0	0.8 ± 0.2	34.8 ± 7.2
**Blocking**	7.6 ± 0.8	20.8 ± 2.4	10.2 ± 2.2	1.6 ± 0.4	6.7 ± 4.0
**120 h p.i.**					
**U87MG**	34.1 ± 2.3	14.1 ± 3.2	5.8 ± 1.0	0.7 ± 0.1	45.2 ± 9.0
**Blocking**	9.9 ± 1.2	23.1 ± 2.3	6.7 ± 1.1	1.3 ± 0.4	7.7 ± 1.6

To further investigate uPA specificity of ^89^Zr-Df-ATN-291, “blocking” experiments were conducted with 1 mg (~40 mg/kg) of unlabeled ATN-291 injected into tumor-bearing mice (*n* = 3) at 2 h before the administration of ^89^Zr-Df-ATN-291. The introduction of unlabeled ATN-291 as a “blocking” dose reduced the tumor uptake of ^89^Zr-Df-ATN-29 at all time points examined (*p* < 0.01) – the uptake was 3.0 ± 0.8, 5.8 ± 1.1, 7.6 ± 0.8, and 9.9 ± 1.2%ID/g at 2, 24, 72, and 120 h p.i., respectively (Figure 2B, 2C and 2D, Table 1). Slightly higher radioactivity in the blood was observed in the “blocking” group at 2 h p.i., whereas liver uptake was significantly higher at all time points. An interesting fact in the blocking group is that tumor uptake continues to increase between 72 and 120 h p.i., while tumor uptake declines slightly at the same time frame in the non-blocking group. One possible explanation here is that tumor can have some “compensating” effect for more uPA production at this blocking dose since sufficient amount of ATN-291 combines with uPA and impairs with its biological functions in tumor.

### PET imaging of ^89^Zr-Df-ATN-291 in different tumor models

**Figure 3:**
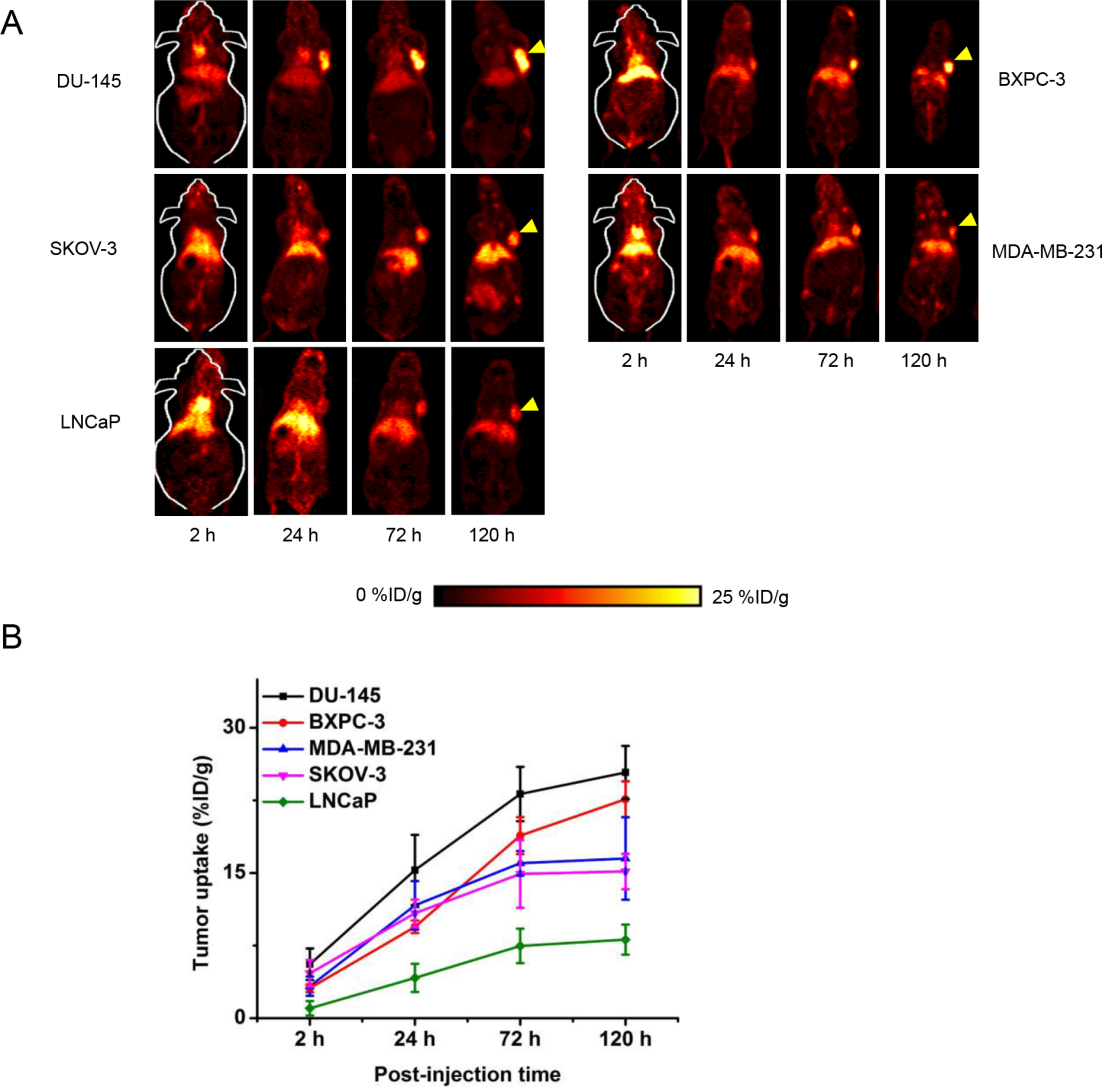
Serial PET studies in different tumor types (**A**) Representative PET images (coronal slice) of mice bearing different tumor types at 2, 24, 72, and 120 h p.i. (*n* = 4 per tumor type). Yellow arrowheads indicate the location of tumors in each mouse. (**B**) Time-activity curves of different tumor types post-injection of ^89^Zr-Df-ATN-291.

In order to demonstrate the universal applicability of ^89^Zr-Df-ATN-291 in different cancer types and correlate its tumor uptake with uPA expression level *in vivo*, we expanded the PET studies in five more tumor models, including BxPC-3 (pancreatic adenocarcinoma), DU-145 (prostate carcinoma, derived from brain metastasis), SKOV-3 (ovarian adenocarcinoma), LNCaP (prostate adenocarcinoma, derived from lymph node metastasis), and MDA-MB-231 (triple-negative breast adenocarcinoma, derived from pleural metastasis). Based on the PET images (Figure 3A), the uptake of ^89^Zr-Df-ATN-291 in normal organs (e.g. liver, blood, muscle etc.) from various tumor-bearing mice followed the same trend as the uptake in U87MG tumor-bearing mice, without noticeable differences (Table 2). The time-activity curves of ^89^Zr-Df-ATN-291 in different tumors are shown in Figure 3B and spanned a wide range (9–30%ID/g). Based on the PET imaging results, U87MG, DU-145 and BxPC-3 tumors had the highest ^89^Zr-Df-ATN-291 uptake (> 20%ID/g at 120 h p.i.), MDA-MB-231, SKOV-3 tumors had medium ^89^Zr-Df-ATN-291 uptake (15–20%ID/g at 120 h p.i.), while LNCaP tumors possessed low ^89^Zr-Df-ATN-291 uptake (< 10%ID/g at 120 h p.i.).

**Table 2:** Tissue uptake of ^89^Zr-Df-ATN-291 in different tumor-bearing mice (n = 4 for all tumor types, except LNCaP (n = 3))

	Tumor (%ID/g)	Liver (%ID/g)	Blood (%ID/g)	Muscle (%ID/g)	Tumor-to-muscle
**2 h p.i.**					
**DU-145**	5.6 ± 1.6	11.6 ± 1.0	22.0 ± 3.8	0.7 ± 0.1	8.1 ± 3.1
**BxPC-3**	3.1 ± 0.4	17.5 ± 6.5	17.6 ± 1.0	0.9 ± 0.5	5.3 ± 4.4
**MDA-MB-231**	3.3 ± 1.0	21.0 ± 3.9	26.4 ± 5.1	1.1 ± 0.3	3.8 ± 2.4
**SKOV-3**	4.6 ± 1.4	18.4 ± 2.7	20.0 ± 5.2	1.1 ± 0.3	4.4 ± 0.2
**LNCaP**	1.0 ± 0.7	19.1 ± 2.4	25.2 ± 4.3	1.0 ± 0.3	1.5 ± 0.6
**24 h p.i.**					
**DU-145**	15.3 ± 3.6	11.6 ± 2.5	13.4 ± 2.1	0.6 ± 0.2	26.6 ± 12.9
**BxPC-3**	9.4 ± 0.6	16.7 ± 6.8	9.2 ± 0.8	1.3 ± 0.3	7.4 ± 1.8
**MDA-MB-231**	11.7 ± 2.5	15.9 ± 2.6	12.7 ± 2.5	1.1 ± 0.5	9.4 ± 1.5
**SKOV-3**	10.8 ± 1.4	14.7 ± 0.4	11.3 ± 4.7	1.0 ± 0.3	9.2 ± 2.2
**LNCaP**	4.2 ± 1.5	18.4 ± 3.7	12.4 ± 2.1	1.2 ± 0.2	3.3 ± 0.8
**72 h p.i.**					
**DU-145**	23.2 ± 2.8	12.1 ± 3.2	8.0 ± 0.9	0.5 ± 0.03	44.9 ± 4.8
**BxPC-3**	18.9 ± 1.9	16.4 ± 5.1	6.0 ± 1.2	1.1 ± 0.2	21.3 ± 3.8
**MDA-MB-231**	15.0 ± 1.3	16.0 ± 3.7	7.5 ± 1.8	1.5 ± 0.2	11.4 ± 2.1
**SKOV-3**	14.9 ± 3.5	14.7 ± 0.4	7.2 ± 3.0	1.3 ± 0.6	9.2 ± 2.4
**LNCaP**	7.5 ± 1.8	15.7 ± 3.6	6.4 ± 1.3	0.9 ± 0.3	8.9 ± 1.3
**120 h p.i.**					
**DU-145**	25.4 ± 2.7	12.0 ± 3.2	4.7 ± 0.4	0.5 ± 0.05	47.9 ± 10.3
**BxPC-3**	22.6 ± 1.9	16.8 ± 6.1	4.6 ± 1.5	0.9 ± 0.3	25.3 ± 4.3
**MDA-MB-231**	16.5 ± 4.3	16.8 ± 4.3	4.9 ± 0.7	1.0 ± 0.4	13.4 ± 3.2
**SKOV-3**	15.2 ± 1.8	16.5 ± 2.6	4.5 ± 0.9	1.3 ± 0.4	10.1 ± 3.0
**LNCaP**	8.1 ± 1.5	12.4 ± 2.7	4.6 ± 1.2	0.9 ± 0.2	9.4 ± 1.2

### Biodistribution studies

**Figure 4:**
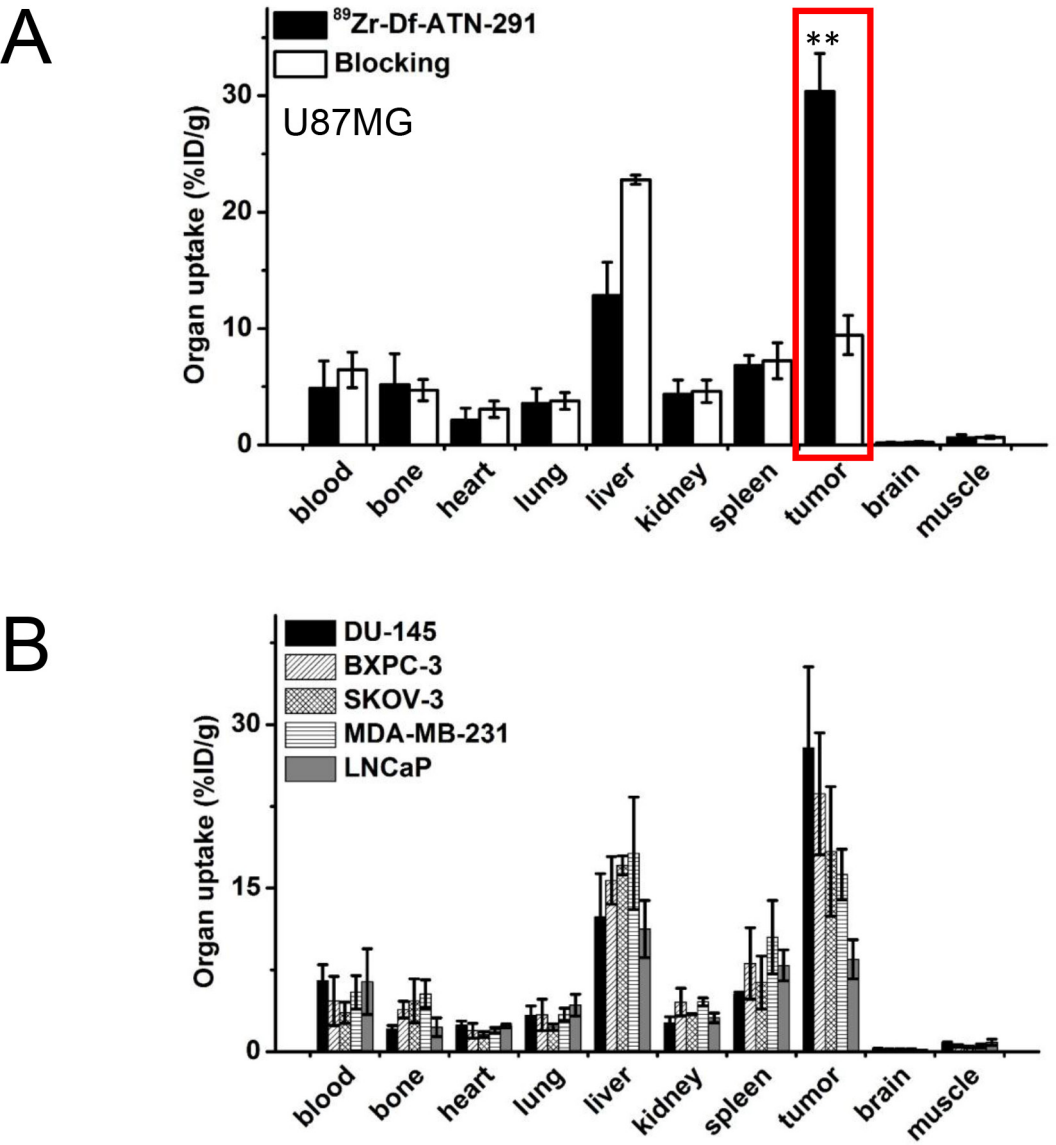
(**A**) Biodistribution of ^89^Zr-Df-ATN-291 alone and ^89^Zr-Df-ATN-291 with blocking in U87MG tumor-bearing mice at 120 h post-injection. Tracer uptake in tumor is highlighted. (**B**) Biodistribution of ^89^Zr-Df-ATN-291 in mice bearing different tumor types at 120 h post-injection.

All mice were euthanized after the terminal PET scans at 120 h p.i. for biodistribution studies to corroborate with the PET data. Tumor, blood, liver, and spleen from all the tumor-bearing mice had significant radioactivity accumulation at 120 h p.i., as expected since a radiolabeled antibody typically has a long circulation half-life and hepatic clearance (Figure 4). Minimal uptake (< 4.5%ID/g for all mice) in kidney was also detected, an observation likely attributed to a partial degradation of ATN-291 over time (into low MW species for renal clearance) and breaking of the chemical bond between Df and ATN-291 (^89^Zr-Df can also undergo renal clearance, and since ^89^Zr-Df chelate is extremely stable [logK > 40], transchelation of ^89^Zr from ^89^Zr-Df-ATN-291 may not be the leading reason). All types of tumor-bearing mice showed a similar ^89^Zr-Df-ATN-291 biodistribution profile in all major organs/tissues except for tumor. These studies demonstrate that an excellent tumor contrast can be achieved in U87MG tumor-bearing mice with a tumor-to-muscle ratio of 48.4 ± 12.3 at 120 h p.i. (*n* = 4).

### Histology and western blotting

**Figure 5:**
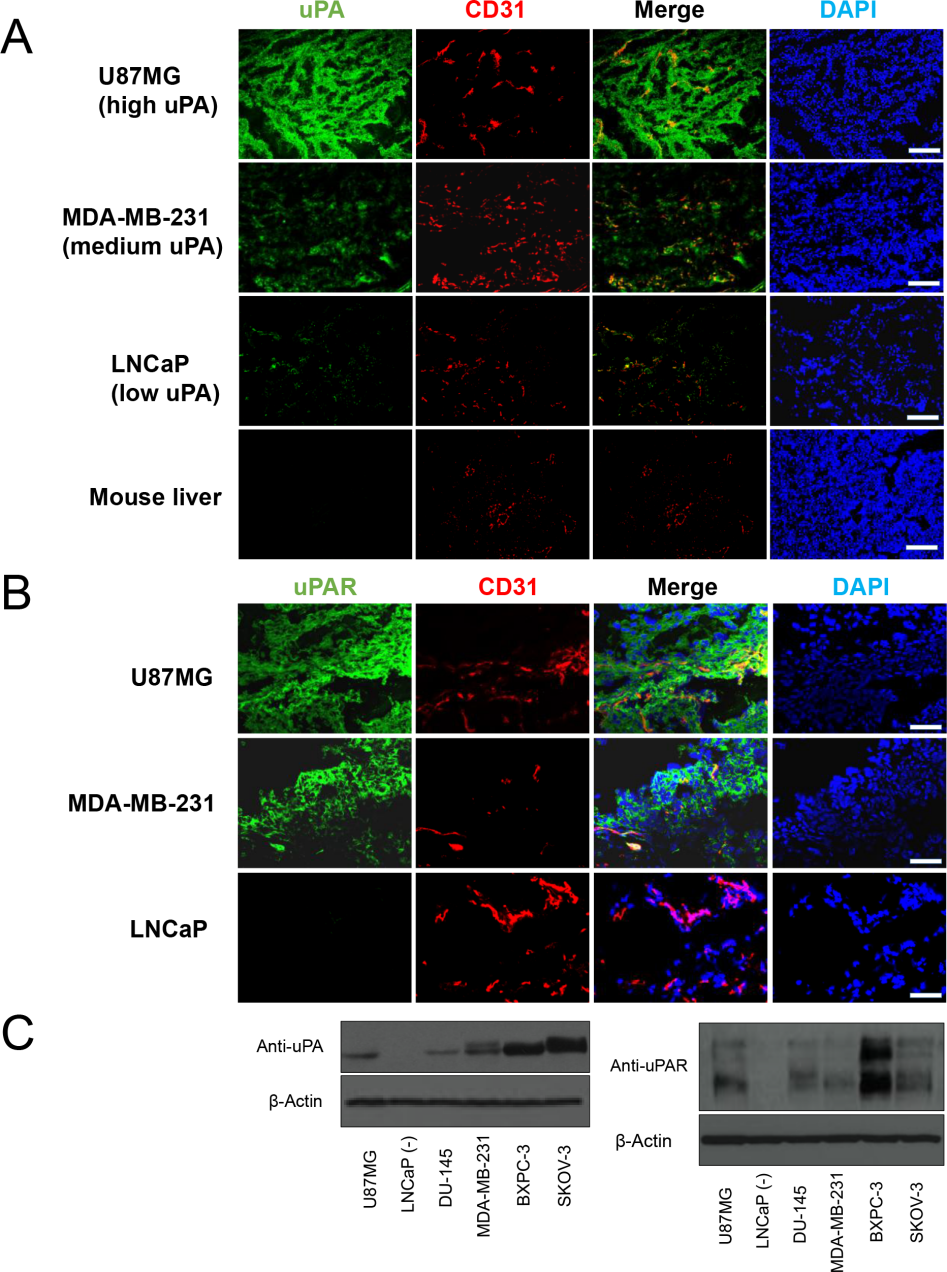
(**A**) Immunofluorescence uPA and CD31 staining of the U87MG (high uPA), MDA-MB-231 (medium uPA), LNCaP (low uPA), and liver sections. ATN-291 and FITC-labeled rabbit anti-mouse IgG were used for uPA staining (green). Subsequently, tissue slices were stained with rat anti-mouse CD31 antibody and Cy3-labeled donkey anti-rat IgG (red). DAPI was used to indicate the location of cell nuclei (blue). All images were acquired under the same conditions and displayed on the same scale. Scale bar: 50 μm. (**B**) Immunofluorescence uPAR and CD31 staining in the U87MG, MDA-MB-231, and LNCaP. Scale bar: 25 μm. (**C**) Western blot of uPA and uPAR was also carried out in lysate of tumors used in PET studies. Twenty μg of total tumor tissue protein was loaded per lane.

Immunofluorescence uPA/CD31 staining revealed that uPA expression was prominent on both tumor cells and the tumor vasculature of U87MG (high uPA), and this expression was substantially weaker but still observable in MDA-MB-231 (medium uPA). Only a small amount of uPA existence was identified in LNCaP tumors. A very important characteristic for the expression patterns of uPA in all these tumor slides was that their distributions were all heterogenous, even within the same tumor (Figure 5A). On the other hand, no uPA existence was seen in liver, which confirmed that the presence of ^89^Zr-Df-ATN-291 in liver was primarily due to non-specific absorption (e.g. from capture by Kupffer cells). In comparison, the expression of uPAR in U87MG was almost equally strong compared with that from MDA-MB-231, while very low level of uPAR expression was observed in LNCaP tumors (Figure 5B). The expression profile of uPA and uPAR obtained from Western blot analysis was consistent with the immunohistological observations (Figure 5C). From these results, we can conclude that the tumor uptake of ^89^Zr-Df-ATN-291 was truly relevant to uPA and uPAR abundance.

## DISCUSSION

The universal overexpression of uPA and uPAR in a variety of tumors is proved to be associated with cancer progression particularly metastasis. This observation has led to rapid development of anti-cancer therapeutics based on targeting of the uPA/uPAR system [6, 27]. In order to meet this fast pace of therapeutic development, different molecular imaging probes have been designed to visualize uPA/uPAR expression *in vivo* with more sensitivity and reliability, providing guidance on evaluation of a specific therapeutic. Radiolabeled AE105, for example, is one outstanding candidate among these imaging probes – it can be used for detection of multiple cancer types [13], and recently ^64^Cu-labeled AE105 has been successfully used for cancer aggressiveness evaluation in patients [16]. Due to encouraging results from these studies, a phase 2 clinical trial is planned with patients being recruited for pre-operative staging of breast cancer by ^68^Ga-NOTA-AE105 PET/CT (NCT02681640 from clinicaltrials.gov). However, one potential limitation from AE105 is that it binds to the uPA binding site in uPAR and therefore either that site must remain unoccupied or depend on AE105 disassociation from uPA for binding. This will likely result in a relatively lower signal-to-noise ratio especially given the typically short plasma half-life of a peptide. Thus, we sought to use a uPA-targeted approach in this study to improve image contrast during PET cancer imaging.

Development of imaging agents against uPA has been a relatively underexplored area. Although, small molecule-based uPA ligands usually possess nanomolar affinity against uPA *in vitro*, they typically demonstrate a comparatively low *in vivo* tumor uptake [19]. The clinical translation of these molecules can be hampered by poor specificity and off-target effects *in vivo*. The good performance of ^111^In-labeled antibody U33 in prostate tumor imaging encouraged us to explore antibodies as contrast agents for *in vivo* uPA targeting [20]. Compared with other uPA-targeted antibodies, ATN-291 used in this study is unique in at least three aspects: it has an extremely high affinity for its target (K_d_: ~ 0.5 nM) [12], it does not interfere with uPA/uPAR binding after it binds to uPA, and its binding to uPA is not competed by PAI-1 [6]. These unique benefits make it readily usable for tumor imaging. One important factor in utilization of an antibody as an imaging agent is to maintain its antigen binding affinity and specificity. The reaction ratio of 10 Df per ATN-291 was chosen to balance the radiolabeling yield and potential loss of affinity. Such a low chelator/antibody ratio can essentially avoid the loss of immunoreactivity, as confirmed by flow cytometry and competitive binding assay (Figure 1). To further maintain the affinity of ATN-291, site-specific labeling (e.g. via cysteine residues or glycans on antibody) will be pursued in the future [28].

^89^Zr was adopted as the imaging label in this study. Over the last decade, mounting bench and bedside data have suggested a promising future for ^89^Zr-based immunoPET in the management of cancer patients [29]. The choice of deferoxamine as the chelator for ^89^Zr is attractive because it has been safely adopted for clinical usage for many years, and no obvious toxicity/immunology responses directed against Df have been reported [30]. *In vivo* stability of radiometal-labeled antibodies always causes concerns. To confirm that the tumor uptake of ^89^Zr-Df-ATN-291 visualized by PET imaging was indeed uPA-specific, various control experiments (e.g. a blocking study) and *in vitro/ex vivo* experiments (e.g. FACS, microscopy, and histological examination) were performed for validation purposes. Based on the available literature data, the ^89^Zr-Df conjugate is very stable *in vivo* [21]. Therefore, the key to *in vivo* stability and antigen binding affinity of an ^89^Zr-based antibody tracer lies in the stability of the Df-antibody conjugate. We have confirmed in a previous study that Df-conjugated antibody could maintain its binding affinity/specificity after incubation with mouse serum for up to 7 days [23]. In this study, we consider the stability of ^89^Zr-Df-ATN-291 very satisfactory from the observation of very low radioactivity retained in kidneys or bones (indicates the detachment of ^89^Zr or partial degradation of ^89^Zr-Df-ATN-291) as late as 120 h p.i.

One potential limitation of this study is that subcutaneous tumor models in mice are used during imaging applications, which may not represent the true developmental nature of each cancer type. However, we should clarify that the primary goal of the current study was to validate the usefulness and applicability of ^89^Zr-Df-ATN-291 as a universal cancer imaging agent. For future studies, other more relevant cancer models, for example patient-derived xenograft (PDX) models (with better preserved tumor heterogeneity and therapy relevance) [31], can be used to further reveal the benefits of this antibody-based PET agent. Here, potent and persistent uptake was shown for ^89^Zr-Df-ATN-291 in different types of tumors. Specifically for glioblastoma U87MG, an impressive tumor-to-muscle ratio of 45.2 ± 9.0 (with absolute tumor uptake of 34.1 ± 2.3%ID/g, Table 1) was achieved for ^89^Zr-Df-ATN-291 at 120 h post-injection. This optimal distribution profile provides the pre-requisite for ATN-291 to be conjugated with therapeutic radionuclides, for radioimmunotherapy (RAIT) of cancer. ^177^Lu or ^90^Y can be selected as optimal radiolabels for this type of application in the future [32].

ATN-291 demonstrated strong and specific binding to the kringle domain of uPA, and our study has demonstrated that *in vivo* accumulation of ^89^Zr-Df-ATN-291 in tumors not only showed good correlation with uPA expression level within the tumor (Figure 5), but also was related to uPAR abundance. We believe that this can be partially explained by how ATN-291 interacts with uPA/uPAR: since ATN-291 appears to trigger internalization (via uPAR) when it binds to uPA irrespective of whether PAI-1 is present [6], distinct spatial/temporal distribution profiles of uPA and uPAR in different tumors can complicate the cellular intake efficiency of ATN-291. Also, we are uncertain at this time whether there exists other “modulators” inside certain tumors to tune the interaction potency between ATN-291/uPA complex and uPAR. Although detailed mechanisms remains to be elucidated and is beyond the scope of this research report, we still believe that ^89^Zr-Df-ATN-291 can serve as a useful imaging tool for both cancer (metastasis) detection and evaluation of a given uPA/uPAR-targeted treatment, which we consider as clinically useful.

Similar to other antibodies, the circulation time of ^89^Zr-Df-ATN-291 is relatively long (11.9 ± 3.5 h, Supplementary Figure S1). At the same time, slow tumor accumulation and high background signal in the mononuclear phagocyte system (MPS, e.g. liver), are also inherent limitations for ^89^Zr-Df-ATN-291 (e.g. in U87MG glioblastoma-bearing mice, tumor-to-liver ratio is 2.5 ± 0.4 at 120 h p.i. (*n* = 4)). This can impose radiotoxicity on normal organs when radiolabeled ATN-291 is adopted for RAIT of cancer. One possible solution is to adjust the antibody dose and/or dose schedule to minimize this cumulative radiation (e.g. pretreatment with a large dose of unlabeled ATN-291) [33], which will be one of our future research focus. With proven good specificity for the uPA/uPAR signaling, another more suitable clinical application for ^89^Zr-Df-ATN-291 is that it may be used as a screening agent to identify uPA/uPAR profiles in cancer patients and select the appropriate patient population to benefit more from a given uPA-targeted therapeutic.

## MATERIALS AND METHODS

### Chemicals

ATN-291 was manufactured by SDIX [12]. Fluorescein isothiocyanate (FITC)- and Cy3-labeled secondary antibodies used in flow cytometry or histology were purchased from Jackson Immunoresearch Laboratories, Inc. (West Grove, CA). Anti-uPA antibody, anti-uPAR antibody and anti-β-actin antibody (conjugated with horseradish peroxidase [HRP]) used in Western blotting were both purchased from Abcam (Cambridge, MA, USA). Secondary HRP antibodies were purchased from Jackson ImmunoResearch (St. Louis, MO, USA). p-SCN-Bn-Df (i.e. *p*-isothiocyanatobenzyl-desferrioxamine B) was acquired from Macrocyclics, Inc. (Cat #: B-705, Dallas, TX). Chelex 100 resin (50 – 100 mesh) was purchased from Sigma-Aldrich (St. Louis, MO). Buffers used in this study were prepared from Millipore-grade water and pre-treated with Chelex 100 resin to ensure that the aqueous solution was free of heavy metals. Size exclusion PD-10 columns were purchased from GE Healthcare (Piscataway, NJ). All other chemicals were purchased from Thermo Fisher Scientific (Fair Lawn, NJ).

### Conjugation and radiolabeling of ATN-291

Desferrioxamine (Df) conjugation onto ATN-291 was carried out according to previously reported procedures with minor modifications [21, 23]. Briefly, a reaction molar ratio of 1:10 was chosen between p-SCN-Bn-Df and ATN-291 – the mixture was kept at 37°C and pH of 9.0 (adjusted with 0.1 N sodium carbonate) for 1 h. Immediately after incubation, Df-ATN-291 was purified using PD-10 columns with 0.25 M sodium acetate (pH 5.5, supplemented with 5 mg/mL gentisic acid) as the mobile phase.

^89^Zr-oxalate was produced with a 11.4-MeV CTI RDS 112 cyclotron via a ^89^Y(p,n)^89^Zr reaction [23]. The ^89^Zr produced has a specific activity of 195–497 MBq/μg after elution from the hydroxamate resin. For radiolabeling of ATN-291, ^89^Zr-oxalate (111–185 MBq, volume ~ 200 μL) was neutralized with 2 M aqueous sodium carbonate (~90 μL) and reacted with Df-ATN-291 in the ratio of 30 μg Df-ATN-291 per 37 MBq of ^89^Zr. The total reaction volume was finalized into 1.5 mL with 0.5 M HEPES buffer (pH 7.1–7.3) and the final reaction pH was adjusted to the range of 6.8–7.2. The mixture was incubated for 1 h at room temperature with constant shaking at 350 rpm. ^89^Zr-Df-ATN-291 was also purified by PD-10 with 0.25 M sodium acetate + 5 mg/mL gentisic acid (pH 5.5) as the mobile phase. The radiolabeling efficiency and product purity was determined by instant thin layer chromatography (ITLC) strips (Biodex) using 20 mM citric acid (pH 4.9–5.1) as ITLC eluent. The radioactive fractions containing ^89^Zr-Df-ATN-291 were collected and passed through a 0.2 μm syringe filter prior to *in vivo* administration.

### Competitive cell binding assay

The binding affinity of ATN-291 and Df-ATN-291 to cellular uPA was evaluated via a displacement cell-binding assay using ^89^Zr-Df-ATN-291 as the radioligand [34]. Briefly, 1 × 10^5^ U87MG human glioblastoma cells (uPA^+^) were seeded to each well of 96-well multiscreen DV plates (Millipore, Billerica, MA) and incubated with ^89^Zr-Df-ATN-291 (~50,000 cpm per well) in the presence of increasing concentrations of ATN-291/Df-ATN-291 (range, 0.1 nM–5 μM). The final volume was adjusted to 200 μL per well. After a 2 h incubation at 37°C, the liquid was removed by vacuum and rinsed three times with cold phosphate buffered saline (PBS) containing 0.1% bovine serum albumin (BSA). After drying, the PVDF filter from each well was collected and counted in an automated γ-counter (WIZARD^2^, Perkin-Elmer). IC_50_ values for both ATN-291 and Df-ATN-291 were calculated from competitive binding curves in GraphPad Prism software (v 6.02, GraphPad Software Inc.). All data points were carried out in triplicate.

### Flow cytometry and fluorescence microscopy examination

The uPA-targeting efficacy of ATN-291/Df-ATN-291 was also assessed with fluorescence-activated cell sorting (FACS) analysis in U87MG cells. Trypsinized cells were suspended in cold PBS with 2% BSA at a concentration of 2 × 10^6^ cells/mL. After incubation with ATN-291 or Df-ATN-291 (5 μg/mL) for 30 min at room temperature, these cells were washed three times with cold PBS. Second incubation with FITC-labeled rabbit anti-mouse IgG (1 μg/mL) was carried out for 30 min at room temperature. Subsequently, the cells were washed thrice with cold PBS and analyzed using a BD LSR Fortessa four-color analysis cytometer, which is equipped with 488nm and 633nm lasers (Becton-Dickinson, San Jose, CA). Fluorescence distributions of the cells were computed using FlowJo analysis software (vX.0.7, Tree Star, Inc., Ashland, OR). U87MG cells, along with LNCaP cells (uPA^−^) [20] were also examined under a Nikon A1 confocal microscope with a magnitude of 200× to validate the FACS results.

### Tumor-bearing mouse model

All animal studies were conducted under a protocol (PRO00006023) approved by the University Committee on Use and Care of Animals (UCUCA) at University of Michigan. Tumors were established by subcutaneous injection of 5 × 10^6^ of U87MG, BxPC-3, DU-145, LNCaP, and SKOV-3, or 2 × 10^6^ of MDA-MB-231 cells suspended in 100 μL of a 1:1 mixture of PBS and Matrigel (BD Biosciences, Franklin Lakes, NJ) into the front flank of nude mice (male for DU-145 and LNCaP, female for the rest of tumor types) purchased from Charles River Laboratories. The tumor sizes were monitored every other day and the mice were subjected to *in vivo* experiments when the tumor diameter reached 5–8 mm (typically 4–6 weeks after inoculation).

### PET imaging and biodistribution studies

PET scans were performed using a preclinical Inveon microPET/CT (Siemens Medical Solutions USA, Inc.). Each tumor-bearing mouse was injected with 5–10 MBq of ^89^Zr-Df-ATN-291 into its tail vein and subjected to static PET scans (40 million events per scan) at various time points post-injection (p.i.). The images were reconstructed using a three-dimensional ordered subset expectation maximization (3D-OSEM) algorithm, with no attenuation or scatter correction. For each microPET scan, Inveon Research Workshop (IRW, v4.2.0.8) was used to superimpose three-dimensional (3D) regions-of-interest (ROIs) on the tumor and major organs in the decay-corrected whole-body images. By adopting a tissue density of 1 g/mL, the radioactivity in each ROI volume was converted to MBq/g using a conversion factor and then divided by the total administered radioactivity to obtain a percentage of injected dose per gram of tissue (%ID/g) for each organ/tissue.

Biodistribution studies were carried out to confirm that the quantitative tracer uptake values based on PET imaging truly represented the radioactivity distribution in tumor-bearing mice. After the last PET scans at 120 h p.i., mice were euthanized and blood, tumors, and major organs/tissues were collected and wet-weighed. The radioactivity in each collected sample was measured using a WIZARD^2^ automatic gamma-counter (Perkin-Elmer) and recorded as %ID/g (mean ± SD). The tumors were also frozen for histological analysis.

### Histology

Frozen tissue slices of 6 μm thickness were fixed with cold acetone for 10 min and air dried in the laboratory for 30 min. After rinsing with PBS and blocking with 10% donkey serum for 30 min at room temperature, the slices were incubated with ATN-291 (5 μg/mL) for 1 h at room temperature and visualized using FITC-labeled rabbit anti-mouse secondary antibody. The tissue slices were also stained for endothelial marker CD31. After washing with PBS, the tissue slices were incubated with rat anti-mouse CD31 antibody (Clone: MEC13.3, BD Biosciences, 1:50 dilution with PBS) for 1 h, followed by Cy3-labeled donkey anti-rat IgG (2 μg/mL) for 30 min. Cell nuclei were visualized by DAPI contained in the mounting medium. Separate batch of tissue slices were also stained for uPAR/CD31/DAPI following similar procedures (uPAR was stained by an Abcam anti-uPAR antibody [Cat # ab52327] following manufacturer's instructions). All images were obtained with a Nikon A1 confocal microscope at the magnitude of 100 ×.

### Western blotting

Tumor samples were collected and snap-frozen in liquid nitrogen. Frozen tumor tissues were homogenized in ice-cold RIPA buffer supplemented with protease inhibitors (Complete Protease Inhibitor Cocktail, Roche, Basel, Switzerland) and phosphatase inhibitors (PhosSTOP, Roche, Basel, Switzerland). Concentration of protein was determined using Lowry assays (Bio-Rad, Hercules, CA) and equal amount of whole tissue protein lysate was loaded in each lane and resolved using 4–12% gradient Bis-Tris gel (Invitrogen, CA). Proteins were transferred to 0.2 μm nitrocellulose membrane (Invitrogen, CA). Membranes were incubated overnight at 4°C with primary antibodies after blocking, followed by incubation with appropriate horseradish peroxidase (HRP)-conjugated secondary antibody at room temperature for one hour. ECL-Plus was used to detect the activity of peroxidase according to the manufacturer's protocol (Amersham Pharmacia, Uppsala, Sweden). The films were scanned using grayscale mode after their development.

### Statistical analysis

Data are presented in the format of mean ± SD. Means were compared using Student's *t*-test. *P* values < 0.05 were considered statistically significant.

## CONCLUSIONS

Herein we report the characterization and *in vivo* investigation of ^89^Zr-labeled ATN-291, a monoclonal antibody against uPA, in different tumor models with various expression levels of uPA and uPAR. ^89^Zr-Df-ATN-291 exhibited prominent and persistent uptake in these tumors, and the uptake values obtained from PET correlate with tumor uPA and uPAR expression. PET imaging can evaluate the pharmacokinetics, tumor targeting efficacy, and dose optimization of ATN-291, preparing it for future uPA-targeted cancer therapy, patient screening, and image-guided tumor surgery in clinics.

## SUPPLEMENTARY MATERIALS AND FIGURE



## References

[1] Vicente-Manzanares M, Horwitz AR (2011). Cell migration: an overview. Methods Mol Biol.

[2] Dass K, Ahmad A, Azmi AS, Sarkar SH, Sarkar FH (2008). Evolving role of uPA/uPAR system in human cancers. Cancer Treat Rev.

[3] McMahon BJ, Kwaan HC (2015). Components of the plasminogen-plasmin system as biologic markers for cancer. Adv Exp Med Biol.

[4] Schmitt M, Harbeck N, Brunner N, Janicke F, Meisner C, Muhlenweg B, Jansen H, Dorn J, Nitz U, Kantelhardt EJ, Thomssen C (2011). Cancer therapy trials employing level-of-evidence-1 disease forecast cancer biomarkers uPA and its inhibitor PAI-1. Expert Rev Mol Diagn.

[5] Mazar AP (2008). Urokinase plasminogen activator receptor choreographs multiple ligand interactions: implications for tumor progression and therapy. Clin Cancer Res.

[6] O'Halloran TV, Ahn R, Hankins P, Swindell E, Mazar AP (2013). The many spaces of uPAR: delivery of theranostic agents and nanobins to multiple tumor compartments through a single target. Theranostics.

[7] Werb Z (1997). ECM and cell surface proteolysis: regulating cellular ecology. Cell.

[8] Friedl P, Wolf K (2003). Tumour-cell invasion and migration: diversity and escape mechanisms. Nat Rev Cancer.

[9] Ellis V, Behrendt N, Dano K (1991). Plasminogen activation by receptor-bound urokinase. A kinetic study with both cell-associated and isolated receptor. J Biol Chem.

[10] Mazar AP, Ahn RW, O'Halloran TV (2011). Development of novel therapeutics targeting the urokinase plasminogen activator receptor (uPAR) and their translation toward the clinic. Curr Pharm Des.

[11] Ahn RW, Chen F, Chen H, Stern ST, Clogston JD, Patri AK, Raja MR, Swindell EP, Parimi V, Cryns VL, O'Halloran TV (2010). A novel nanoparticulate formulation of arsenic trioxide with enhanced therapeutic efficacy in a murine model of breast cancer. Clin Cancer Res.

[12] Zhang Y, Kenny HA, Swindell EP, Mitra AK, Hankins PL, Ahn RW, Gwin K, Mazar AP, O'Halloran TV, Lengyel E (2013). Urokinase plasminogen activator system-targeted delivery of nanobins as a novel ovarian cancer therapy. Mol Cancer Ther.

[13] Li D, Liu S, Shan H, Conti P, Li Z (2013). Urokinase plasminogen activator receptor (uPAR) targeted nuclear imaging and radionuclide therapy. Theranostics.

[14] Juhl K, Christensen A, Persson M, Ploug M, Kjaer A (2016). Peptide-Based Optical uPAR Imaging for Surgery: *In Vivo* Testing of ICG-Glu-Glu-AE105. PLoS One.

[15] Persson M, Nedergaard M, Brandt-Larsen M, Skovgaard D, Jorgensen JT, Michaelsen SR, Madsen J, Lassen U, Poulsen HS, Kjaer A (2016). uPAR is a promising new imaging biomarker in glioblastoma. J Nucl Med.

[16] Persson M, Skovgaard D, Brandt-Larsen M, Christensen C, Madsen J, Nielsen CH, Thurison T, Klausen TL, Holm S, Loft A, Berthelsen AK, Ploug M, Pappot H (2015). First-in-human uPAR PET: Imaging of Cancer Aggressiveness. Theranostics.

[17] LeBeau AM, Sevillano N, King ML, Duriseti S, Murphy ST, Craik CS, Murphy LL, VanBrocklin HF (2014). Imaging the urokinase plasminongen activator receptor in preclinical breast cancer models of acquired drug resistance. Theranostics.

[18] Boonstra MC, van Driel PB, van Willigen DM, Stammes MA, Prevoo HA, Tummers QR, Mazar AP, Beekman FJ, Kuppen PJ, van de Velde CJ, Lowik CW, Frangioni JV, van Leeuwen FW (2015). uPAR-targeted multimodal tracer for pre- and intraoperative imaging in cancer surgery. Oncotarget.

[19] Ides J, Thomae D, Wyffels L, Vangestel C, Messagie J, Joossens J, Lardon F, Van der Veken P, Augustyns K, Stroobants S, Staelens S (2014). Synthesis and *in vivo* preclinical evaluation of an ^18^F labeled uPA inhibitor as a potential PET imaging agent. Nucl Med Biol.

[20] LeBeau AM, Sevillano N, Markham K, Winter MB, Murphy ST, Hostetter DR, West J, Lowman H, Craik CS, VanBrocklin HF (2015). Imaging active urokinase plasminogen activator in prostate cancer. Cancer Res.

[21] Vosjan MJ, Perk LR, Visser GW, Budde M, Jurek P, Kiefer GE, van Dongen GA (2010). Conjugation and radiolabeling of monoclonal antibodies with zirconium-89 for PET imaging using the bifunctional chelate p-isothiocyanatobenzyl-desferrioxamine. Nat Protoc.

[22] Hong H, Zhang Y, Nayak TR, Engle JW, Wong HC, Liu B, Barnhart TE, Cai W (2012). Immuno-PET of tissue factor in pancreatic cancer. J Nucl Med.

[23] Hong H, Severin GW, Yang Y, Engle JW, Zhang Y, Barnhart TE, Liu G, Leigh BR, Nickles RJ, Cai W (2012). Positron emission tomography imaging of CD105 expression with ^89^Zr-Df-TRC105. Eur J Nucl Med Mol Imaging.

[24] Hong H, Yan Y, Shi S, Graves SA, Krasteva LK, Nickles RJ, Yang M, Cai W (2015). PET of follicle-stimulating hormone receptor: broad applicability to cancer imaging. Mol Pharm.

[25] Bu X, Khankaldyyan V, Gonzales-Gomez I, Groshen S, Ye W, Zhuo S, Pons J, Stratton JR, Rosenberg S, Laug WE (2004). Species-specific urokinase receptor ligands reduce glioma growth and increase survival primarily by an antiangiogenesis mechanism. Lab Invest.

[26] Holland JP, Divilov V, Bander NH, Smith-Jones PM, Larson SM, Lewis JS (2010). ^89^Zr-DFO-J591 for immunoPET of prostate-specific membrane antigen expression *in vivo*. J Nucl Med.

[27] Carriero MV, Franco P, Votta G, Longanesi-Cattani I, Vento MT, Masucci MT, Mancini A, Caputi M, Iaccarino I, Stoppelli MP (2011). Regulation of cell migration and invasion by specific modules of uPA: mechanistic insights and specific inhibitors. Curr Drug Targets.

[28] Adumeau P, Sharma SK, Brent C, Zeglis BM (2016). Site-Specifically Labeled Immunoconjugates for Molecular Imaging-Part 1: Cysteine Residues and Glycans. Mol Imaging Biol.

[29] Zhang Y, Hong H, Cai W (2011). PET tracers based on Zirconium-89. Curr Radiopharm.

[30] Borjesson PK, Jauw YW, Boellaard R, de Bree R, Comans EF, Roos JC, Castelijns JA, Vosjan MJ, Kummer JA, Leemans CR, Lammertsma AA, van Dongen GA (2006). Performance of immuno-positron emission tomography with zirconium-89-labeled chimeric monoclonal antibody U36 in the detection of lymph node metastases in head and neck cancer patients. Clin Cancer Res.

[31] Cassidy JW, Caldas C, Bruna A (2015). Maintaining Tumor Heterogeneity in Patient-Derived Tumor Xenografts. Cancer Res.

[32] Bloy N, Pol J, Manic G, Vitale I, Eggermont A, Galon J, Tartour E, Zitvogel L, Kroemer G, Galluzzi L (2014). Trial Watch: Radioimmunotherapy for oncological indications. Oncoimmunology.

[33] Goldenberg DM (2002). Targeted therapy of cancer with radiolabeled antibodies. J Nucl Med.

[34] Luo H, Hernandez R, Hong H, Graves SA, Yang Y, England CG, Theuer CP, Nickles RJ, Cai W (2015). Noninvasive brain cancer imaging with a bispecific antibody fragment, generated via click chemistry. Proc Natl Acad Sci USA.

