# Supplementary material for: Antibody-based PET of uPA/uPAR signaling with broad applicability for cancer imaging
Source: Oncotarget. 2016 Oct 8;7(45):73912–24. doi: 10.18632/oncotarget.12528 (PMC5342023; doi:10.18632/oncotarget.12528)
Supplement: Supplementary file 1 [file oncotarget-07-73912-s001.pdf]

Antibody-based PET of uPA/uPAR signaling with broad applicability for cancer imaging

Supplementary Materials

Circulation half-life measurement by sequential blood sampling

In order to evaluate the circulation half-life of ^{89}Zr-Df-ATN-291, three nude mice were each injected with 5–10 MBq of ^{89}Zr-Df-ATN-291 (similar to the doses used in PET imaging studies). Approximately 5–40 μL of blood was taken from the tail vein of each mouse at different time points post-injection and weighted immediately. The radioactivity in the blood samples was measured using the WIZARD^2 γ-counter (Perkin-Elmer) and calculated as percentage of injected dose per gram of tissue (% ID/g). The values of blood circulation half-life were calculated based on one-phase exponential decay in GraphPad Prism (v 6.02, GraphPad Software). Based on the circulation-radioactivity decay curves (Supplementary Figure S1) in different animals, the circulation half-life of ^{89}Zr-Df-ATN-291 was determined to be 11.9 ± 3.5 h (Mouse 1: 12.82 h; Mouse 2: 14.81 h; Mouse 3: 8.006 h).

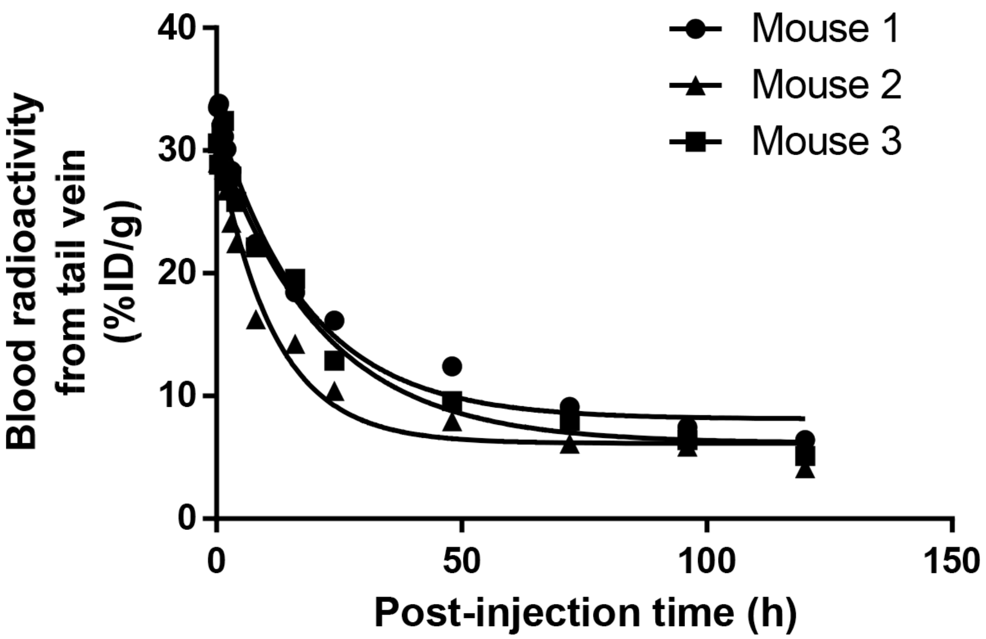

Supplementary Figure S1: Circulation half life calculation for 89Zr-Df-ATN-291 in nude mice (*n* = 3).
